# The Gut–Liver Axis in Pediatric Liver Health and Disease

**DOI:** 10.3390/microorganisms11030597

**Published:** 2023-02-27

**Authors:** Stephanie L. Rager, Melody Y. Zeng

**Affiliations:** 1 Gale and Ira Drukier Institute for Children’s Health, Weill Cornell Medicine, New York, NY 10021, USA; 2 Department of Pediatrics, Weill Cornell Medicine, New York, NY 10021, USA; 3 Immunology and Microbial Pathogenesis Graduate Program, Weill Cornell Medicine, New York, NY 10021, USA

**Keywords:** gut microbiota, pediatrics, hepatobiliary disease, biliary atresia, NAFLD, IBD

## Abstract

There has been growing interest in the complex host–microbe interactions within the human gut and the role these interactions play in systemic health and disease. As an essential metabolic organ, the liver is intimately coupled to the intestinal microbial environment via the portal venous system. Our understanding of the gut–liver axis comes almost exclusively from studies of adults; the gut–liver axis in children, who have unique physiology and differing gut microbial communities, remains poorly understood. Here, we provide a comprehensive overview of common pediatric hepatobiliary conditions and recent studies exploring the contributions of the gut microbiota to these conditions or changes of the gut microbiota due to these conditions. We examine the current literature regarding the microbial alterations that take place in biliary atresia, pediatric non-alcoholic fatty liver disease, Wilson’s disease, cystic fibrosis, inflammatory bowel disease, and viral hepatitis. Finally, we propose potential therapeutic approaches involving modulation of the gut microbiota and the gut–liver axis to mitigate the progression of pediatric liver disease.

## 1. Introduction

### 1.1. The Pediatric Gut Microbiota

The interactions between humans and the bacteria that reside within our gastrointestinal tract are intricately balanced and have evolved to reach a mutually beneficial and sustainable symbiosis [1]. In a healthy individual, the community of microorganisms within the gut, referred to collectively as the gut microbiota, confers resistance against colonization by enteric pathogens, supplies essential nutrients such as vitamins and short-chain fatty acids (SCFAs) by assisting in the digestion of otherwise indigestible complex carbohydrates, and regulates host metabolism and gut–epithelial barrier function [2,3,4]. The gut microbiota is sensitive to and shaped by a variety of host factors, such as age, diet, stress, inflammation, gastrointestinal motility, environmental toxin exposure, gastric acid and bile secretion, and enteric pathogens [5]. Dysbiosis of the gut microbiota, often due to changes in these factors, may contribute to the pathogenesis of a variety of gastrointestinal or systemic disorders.

The first major bacterial colonization of the gut occurs immediately after birth by species that are capable of inhabiting the initially aerobic environment of the immature gastrointestinal tract. These early colonizers include aerotolerant *Bifidobacterium*, microaerophilic *Lactobacillus*, and facultative *Enterobacteriaceae* species. The initial bacterial composition differs between infants delivered vaginally, who are exposed to maternal vaginal and fecal bacteria, and those who are delivered by cesarean section [6,7,8]. Infants born by cesarean section also have delayed intestinal colonization by *Bacteroides* [6,7,8]. Prior to weaning, *Bifidobacterium* and *Lactobacillus* species dominate the healthy infant microbiota, as the primarily available nutrients, including human milk oligosaccharides (HMOs), are derived from breast milk and favor the growth of these aerotolerant anaerobes. Around 6 months of age, the introduction of solid foods consisting of more fiber leads to blooms of fiber-fermenting *Clostridium*, *Akkermansia*, *Bacteroides*, and *Ruminococcus* species and a decrease in *Escherichia* and *Staphylococcus* species [9,10,11]. The gastrointestinal microbial community is subsequently shaped by common childhood illnesses and the use of antibiotics until it stabilizes around three years of age [12]. This progressive temporal colonization of diversifying gut bacterial species within the infant gut is believed to be an important driver of the maturation of the immune system as well as overall metabolism.

### 1.2. The Gut–Liver Axis

**Figure 1:**
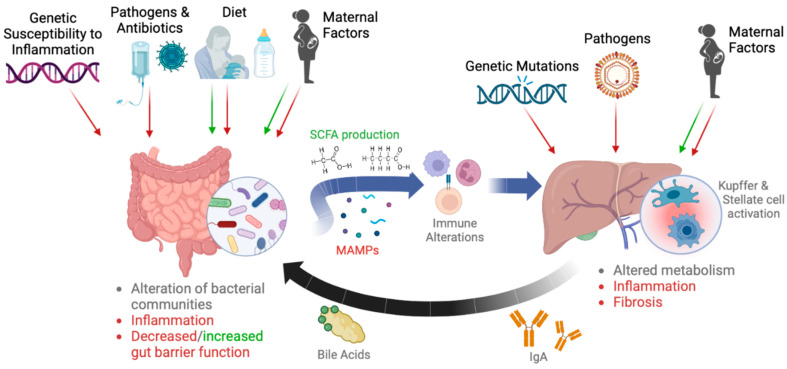
The bidirectional gut–liver axis in children. Various factors, such as diet (breast milk, formula, parenteral nutrition, the transition to solid foods, and type of solid food diet), exposure to common viral and bacterial enteric pathogens, genetic susceptibility to inflammatory conditions such as inflammatory bowel disease, and maternal factors such as diet or metabolic diseases, are known to influence the composition and diversity of the pediatric gut microbiota. While some of these factors, such as breastfeeding or a low-fat maternal diet, may have beneficial effects on the gut environment (indicated in green), others, such as early life antibiotic exposure, have deleterious effects (indicated in red). Microbe-associated molecular patterns (MAMPs) from bacteria themselves as well as bacterial metabolites, such as short-chain fatty acids (SCFAs), then influence the activity of local immune cells. MAMPs eventually reach the liver through the portal vein and may result in hepatic inflammation and altered metabolism, while SCFAs produced by beneficial bacteria have generally anti-inflammatory effects on liver cells. Breakdowns in gut barrier function from enteric infection or inflammation may also lead to the direct translocation of bacteria into the portal system, causing more severe liver inflammation and fibrosis. On the other hand, genetic mutations, viral infection, and maternal factors such as obesity (red arrows) may result in primary hepatobiliary diseases, including Wilson’s disease and pediatric non-alcoholic fatty liver disease. Other maternal factors, such as healthy maternal weight, are protective (green arrow) and may decrease risk of liver disease. Alterations in hepatic IgA and bile acid secretion resulting from inflammation or structural remodeling impact the bacterial communities able to exist within the GI tract, which may lead to gut dysbiosis. Figure created with BioRender.com.

Alterations to the gut microbiota may result in metabolic and immune dysfunction at distal organs, as immune cells and signaling molecules traffic from the gut through the bloodstream. The liver is directly linked with the rest of the gastrointestinal tract through portal venous circulation, allowing for significant gut–liver crosstalk; bacteria-derived signaling molecules make their way from the small intestine and colon into the superior and inferior mesenteric veins, then move into the hepatic circulation through the portal vein (Figure 1) [13]. Here, microbial-associated molecule patterns (MAMPs), such as flagellin and peptidoglycans, from gut commensal bacteria trigger maturation and persistent low-level activation of Kupffer cells (KCs), specialized macrophages in the liver [14]. Exposure of KCs to diverse MAMPs results in increased baseline expression of major histocompatibility complex II by KCs and decreased phagocytic activity, contributing to a relatively tolerogenic KC phenotype and therefore general T cell tolerance. Bacterial lipopolysaccharides (LPS) have also been shown to induce the production of inflammatory cytokines Il-8 and monocyte chemoattractant protein-1 (MCP-1) in hepatic stellate cells (HSCs) as well as upregulate their cell surface expression of intracellular adhesion molecule 1 (ICAM-1) and vascular adhesion molecule 1 (VCAM-1), causing HSCs to proliferate and increase collagen synthesis [15]. In a healthy state, the intestinal barrier, enhanced by antimicrobial peptides, protective mucus, and epithelial tight junctions, prevents the direct translocation of bacteria into portal venous circulation (Figure 1) [16]. In diseased states, compromised barrier function may allow bacteria, bacterial ligands, and bacterial metabolites to pass more easily into the bloodstream and on to the liver, resulting in hepatic inflammation and aggravating any underlying liver pathology (Figure 1) [13]. In turn, the hepatobiliary system also influences the microbial landscape through the production of bile acid (which promotes the growth of bile acid-metabolizing bacteria while inhibiting the growth of bile-sensitive bacteria) and the processing and distribution of secretory IgA, a major component of mucosal immunity and intestinal homeostasis (Figure 1) [17,18].

### 1.3. The Pediatric Liver

Although metabolism is a primary role of the adult liver, in utero, the maternal liver performs necessary metabolic activity for the fetus, while the fetal liver is responsible for connecting the heart to the placental vessels and serving as a site of hematopoiesis prior to the development of bone marrow [19]. After birth, the infant’s first feed delivers increased blood flow to the portal system and the liver begins carrying out its postnatal functions. In the neonate, the liver is especially important for plasma protein synthesis, glucose and fatty acid metabolism, bilirubin metabolism, bile synthesis, and the clearance of toxic by-products [20]. The liver does not fully mature until approximately two years of age, and the neonatal liver contains less than 20% of the hepatocytes found in the adult liver [21]. Premature neonates in particular are susceptible to hypoglycemia, cholestasis, bleeding, poor drug metabolism, and hyperbilirubinemia as a result of incomplete liver development [22]. Beyond the neonatal period, rapid maturation of phase I metabolism takes place in the liver, with peak elimination of drugs occurring during early adolescence, after which plasma clearance begins to slow [23].

Because of their unique physiology and relative lack of lifestyle-related risk factors, children suffer from a very different set of hepatobiliary diseases compared to adults. While the most common causes of liver disease in adults include hepatitis C, alcohol-associated liver disease, and nonalcoholic fatty liver disease, children are more likely to suffer from structural, autoimmune, and congenital hepatobiliary disorders [24]. This means that there are also unique ways in which the pediatric gut microbiota interacts with the liver to potentially exacerbate such conditions.

Here, we provide a broad overview of recent work on the interplay between the gut microbiota and some of the most common hepatobiliary diseases affecting the pediatric population. We also discuss the implications of recent findings within the field and potential targets for future therapies for pediatric hepatobiliary disorders.

## 2. Interactions between the Gut Microbiota and Pediatric Hepatobiliary Disorders (Table 1)

### 2.1. Biliary Atresia

One of the most devastating pediatric hepatobiliary conditions is biliary atresia (BA), a largely idiopathic condition of potential genetic, infectious, or inflammatory etiologies that results in progressive, fibrotic biliary obstruction in the neonatal period. BA progresses to cirrhosis in the absence of curative treatment, with a corresponding survival rate of less than 10% by three years, and it is the most common cause of pediatric end-stage liver disease [25]. Given the severity of BA and relative lack of understanding surrounding its pathophysiology, recent studies have investigated the potential roles of both the maternal and neonatal gut microbiotas in the development and treatment of this multifactorial condition.

One murine study suggests a role for the gut microbiota to influence the susceptibility of neonates to biliary injury [26]. Maternal supplementation with butyrate, a short-chain fatty acid (SCFA) produced by bacteria within the gut, largely protected offspring against bile duct injury in a virally induced model of BA. Neonates from butyrate-fed dams had fecal enrichment of *Firmicutes* and *Bacteroidetes*, a signature that was also found among butyrate-fed dams. Offspring of butyrate-fed dams also had increased fecal concentrations of the metabolites glutamate and glutamine. Direct administration of glutamate to neonates prior to viral induction of BA suppressed activation of immune cells and prevented the development of BA. Overall, these results are consistent with the known protective role of butyrate in promoting mucosal barrier function and decreasing inflammation within the gastrointestinal tract through bacteria-mediated pathways [27]. These data suggest that microbial metabolites, namely butyrate and glutamate, may play a role in the development and severity of biliary atresia and should be further investigated for potential therapeutic purposes as postbiotics.

A human study examined a cohort of 43 infants with all-cause cholestasis, generally defined as any impairment in the excretion of bile resulting in hyperbilirubinemia and/or elevated gamma-glutamyl transferase (GGT). This condition may result from obstructive, infectious, genetic, toxic, or idiopathic etiologies. The authors found that when compared with healthy controls, infants with cholestasis had lower bacterial diversity as measured by the Shannon index and enrichment of *Streptococcus*, *Enterococcus*, *Staphylococcus*, *Megasphaera*, *Phyllobacterium*, and *Megamonas* [28]. Neonates with cholestasis had lower proportions of *Bifidobacterium*, *Bacteroides*, and *Faecalibacterium.* There were also direct pairwise correlations between gut bacterial genera and several measures of hepatic functions, including AST, ALT, and total bilirubin, strengthening the assertion that the gut microbiota plays a direct role in regulating hepatobiliary health. Notably, there were no apparent differences in the diversity or composition of the gut microbiota among infants with different causes of cholestasis, including BA. Another case-control study found that infants with BA had lower diversity, increased abundance of more pathogenic genera such as *Streptococcus* and *Klebsiella*, and decreased numbers of potentially beneficial butyrate-producing bacteria, such as *Bacteroides* and *Faecalibacterium* [29].

Treatment of BA necessitates surgery, and the current standard of treatment is the Kasai procedure. This operation involves excision of the fibrotic biliary area and the creation of a Roux-en-Y loop to reestablish biliary-enteric continuity and drainage of bile. A recent study examined fecal samples of two BA infants with similar clinical backgrounds but different outcomes following Kasai portoenterostomy; one infant had a good outcome, with resolution of jaundice by six weeks post-op (BA-G), while the other had a poor outcome and ultimately required liver transplantation (BA-P) [30]. The BA-G infant exhibited higher Fisher’s *alpha* diversity compared to the BA-P infant immediately post-operatively, and the BA-P infant exhibited higher abundance of *Enterobacter*, *Bacteroides*, and *Clostridium* genera. This is interesting considering that *Enterobacteriaceae* is a potentially pathogenic taxon also found to be upregulated in patients with cirrhosis and decreased fecal bile acid [31]. It is also noteworthy that that *Bacteroides* would be increased in the infant with poor transplant outcomes, as some species of *Bacteroides* have been shown to have anti-inflammatory properties, in part due to their ability to produce SCFAs [32]. However, it should be noted that in all cases, the immunomodulatory effects of any genus may vary at the species level, and this is therefore one important limitation of genus-level analyses.

When liver cirrhosis is advanced and/or the Kasai procedure has failed, liver transplant becomes necessary to treat BA. Several studies have investigated the potential role for gut microbiota alterations in long-term liver transplant failure [33]. Qin et al. proposed several mechanisms through which changes in the gut microbiota may lead to pediatric post-transplant graft fibrosis, including translocation of pathogen-associated molecular patterns (PAMPs) resulting from increased intestinal permeability in the setting of dysbiosis [33]. These PAMPs, such as lipopolysaccharides, microbial DNA, and lipopeptides, eventually reach the liver via the portal vein, where they trigger an innate immune response leading to fibrogenesis. Decreased production of SCFAs in the setting of dysbiosis is speculated as another potential cause of fibrosis, as SCFAs play an important role in maintaining intestinal barrier integrity and immune regulation [34,35]. Based on these conjectures, several groups have explored the benefits of pre-/probiotic supplementation and fecal microbiota transplants to improve outcomes in liver disease, with promising results. Notably, however, there have been no studies specifically exploring these interventions in pediatric patients undergoing liver transplant [36].

### 2.2. Pediatric Non-Alcoholic Fatty Liver Disease

With the rise in childhood obesity and metabolic disorders in developing countries, there has been an increase in the incidence of pediatric non-alcoholic fatty liver disease (NAFLD), now the most common chronic liver disease in children. As of 2021, the prevalence of pediatric NAFLD was estimated to be between 5 and 10% globally, with higher rates in males than females [37]. Many studies have supported the role of gut dysbiosis in the development or aggravation of metabolic dysfunction, with alterations in gut flora and their metabolites resulting in increased insulin resistance, increased adiposity, and increased fat deposition in the liver [38,39]. It is therefore no surprise that the gut microbiota may play a part in the development and natural progression of pediatric NAFLD, a disease with a multifactorial pathogenesis influenced by obesity, lipid metabolism, and diet.

A case-control study in Finland followed 25 infants over time and found that children who became overweight by age seven had higher levels of *Staph. aureus*, a potentially pro-inflammatory bacteria, and lower levels of *Bifidobacteria*, a classically anti-inflammatory genus, in their stool during infancy compared to those who maintained a healthy weight [40]. Interestingly, infants who are born via cesarean section have been found to have similar alterations in their gut microbiotas compared to those born via vaginal delivery, with lower abundance of *Bifidobacteria* and higher abundance of *Staphylococcus*, *Streptococcus*, and *Clostridium* [41]. In fact, cesarean delivery has been shown to be an early life risk factor for obesity and NAFLD, though infants in the Finland study were matched for delivery mode [42,43].

Another study found that pediatric patients with NAFLD had significant differences in the abundance of multiple genera in their gut when compared to control pediatric patients [44]. A decrease in *Oscillospira* along with an increase in 2-butanone, a volatile organic compound, was a signature of NAFLD onset, while an increase in *Ruminococcus* and *Dorea* predicted NAFLD progression. Interestingly, a *decrease* in fecal 2-butanone has been found in adult patients with NAFLD, though the potential significance of this discrepancy is unclear [45].

Even more compellingly, a randomized controlled trial involving 65 children with obesity and ultrasound findings consistent with NAFLD found that those who received a probiotic (containing two strains of *Lactobacillus* and two strains of *Bifidobacterium)* for three months had decreased mean levels of alanine aminotransferase, aspartate aminotransferase, cholesterol, low-density lipoprotein C, triglycerides, and waist circumference compared to those patients who received a placebo pill [46]. These findings are also consistent with a slightly smaller-scale study in which 22 children with NAFLD who received the VSL#3 probiotic (containing four strains of *Lactobacillus*, three strains of *Bifidobacterium*, and one strain of *Streptococcus*) for four months had improvement in their disease based on liver ultrasound findings compared to children who received a placebo [47]. Most promisingly, a meta-analysis of these aforementioned studies as well as two other randomized control trials including a total of 238 pediatric patients with NAFLD revealed a significant improvement in liver transaminases, total cholesterol, triglyceride levels, and ultrasound findings suggestive of liver steatosis in patients who received probiotics compared to those who did not [48]. However, heterogeneous strains were used in these studies, and long-term benefits of these probiotics for pediatric NAFLD, as well as the underlying mechanisms of protection, remain to be further investigated.

Maternal factors such as obesity are known to play a role in the pathogenesis of pediatric NAFLD, possibly in part by altering the microbiota of offspring. Soderborg et al. compared gnotobiotic mice gavaged with pooled stool from infants born to mothers with obesity to mice gavaged with stool from infants born to mothers without obesity [49]. Mice colonized with gut bacteria from infants born to obese mothers had increased weight gain, endoplasmic reticulum stress, liver macrophage accumulation, and histologic evidence of increased periportal inflammation in the liver (similar to that seen in pediatric patients with NAFLD). Interestingly, these mice also had immune system alterations, with macrophage hypo-responsiveness to LPS and reduced ability to phagocytose *Listeria*, suggestive of potential immunomodulatory mechanisms either underlying or resulting from the phenotypes observed.

### 2.3. Wilson’s Disease

A less common cause of hepatobiliary metabolic dysfunction in children is Wilson’s disease (also known as hepatolenticular degeneration), an autosomal recessive genetic disorder of impaired biliary copper excretion. Over time, this disease results in excessive accumulation of copper in the cornea, brain, and liver, causing oxidative stress and unregulated apoptosis that can result in cirrhosis.

Copper enters bacteria through porins and membrane transporters, such as the copper uptake porter, which allow copper to cross the extracellular membrane [50]. While copper is an essential cofactor for several metabolic processes, it also exhibits some degree of antibacterial activity, resulting in protein damage and cell injury if present in excess [51]. Consistently, animal studies have found copper to have a direct effect on the composition of bacteria that reside within the gut [52]. A high-copper diet resulted in liver injury in male rats stressed with a high-fructose diet and was associated with gut barrier dysfunction and alterations in the gut microbiota; rats who received high-copper diets had an increased *Firmicutes/Bacteroidetes* ratio (seen in obesity), a unique enrichment of *Erysipelotrichaceae* and *Enterobacteriaceae*, and a reduction in *Akkermansia* [53]. In a study of piglets, copper supplementation was associated with significantly altered gut microbial composition compared to controls, increased *E. coli* in the gut, increased *E. coli* resistance to several antibiotics, and altered microbial metabolic functions related to amino acid synthesis, energy metabolism, and protein metabolism [54].

One clinical study compared the gut microbiota of young patients with Wilson’s disease to those of healthy controls [55]. Significant differences in fecal bacterial composition and diversity were found between groups, with a lower Shannon diversity index and ACE richness index of the intestinal flora in patients with Wilson’s disease compared to that of controls. Patients with Wilson’s disease also had increased abundance of *Bacteroidetes* and lower abundance of *Firmicutes*, *Proteobacteria*, and *Fusobacteria* compared to controls. Interestingly, patients with Wilson’s disease were also found to have lower numbers of T lymphocytes and higher numbers of B lymphocytes, NK cells, and circulating levels of IgM compared to controls.

A more recent study analyzing fecal 16S rRNA data from a slightly older cohort of patients (mean age 32) also found evidence of altered gut microbial environments with potential immune implications in patients with Wilson’s disease [56]. Patients with Wilson’s disease had a significantly lower abundance of *Firmicutes* compared to healthy controls and a higher abundance of the opportunistic pathogen *Proteobacteria*. *Firmicutes* play a key role in the breakdown of undigestible carbohydrates and the production of SCFAs, which have key anti-inflammatory effects and promote the differentiation of regulatory T cells. Therefore, the authors postulated that this microbial alteration may promote an increasingly pro-inflammatory environment within the gut of patients with Wilson’s Disease. Additionally, the functional composition of the gut microbiota of patients with Wilson’s disease was analyzed by KEGG and COG pathway analyses, which revealed a decreased abundance of pathways linked to key aspects of host metabolism, including transcription factors, ABC-type transporters, and the metabolism of butanoate, further suggesting the presence of microbe-driven metabolic dysregulation in patients with Wilson’s disease.

Altogether, these data suggest that metabolic dysfunction from Wilson’s disease may inherently lead to alterations in the gut microbiota that further promote inflammation and worsen the underlying disease state. Therefore, it remains to be seen whether therapies that aim to increase the gut microbial diversity of these patients and restore the normal microbial composition will result in improved immune function.

### 2.4. Cystic Fibrosis-Associated Liver Disease (CFLD)

Cystic fibrosis (CF) is another autosomal recessive disease that affects an estimated 1/3000–1/6000 children and results in multisystem dysfunction [57,58]. While the overall severity of disease is determined by the extent of pulmonary involvement, cystic fibrosis-associated liver disease (CFLD) affects an estimated 30% of CF patients and as of 2014 was the third leading cause of death in patients with CF [59]. The pathophysiology of CFLD is not fully understood, but it is hypothesized that over time, impaired biliary secretion from abnormal cystic fibrosis transmembrane conductance regulator (CFTR) proteins in the apical membrane of cholangiocytes and the production of thick, viscous bile with increased free radicals leads to both direct hepatocyte damage and indirect periportal fibrosis as HSCs are activated to produce collagen [60].

Early in the course of the disease, patients with CF present with gastrointestinal involvement including steatorrhea, bloating, constipation, intestinal inflammation, improper absorption of nutrients leading to a failure to thrive, and altered intestinal barrier function [61]. Repeated antibiotic exposure from increased susceptibility to respiratory infections may further perturb the gut microbiota [62]. Additionally, there is increasing evidence to support the existence of a strong gut–lung axis, wherein perturbations of the bacterial community within the gut exacerbates primary lung disease and vice versa [63,64]. Bacterial communities within the gut and lungs of infants with CF have been found to closely mirror one another and shift concordantly over time, with changes in diet resulting in changes in the lung microbiota [65]. Increased intestinal microbial alpha diversity has also been found to positively correlate with increased time to infants’ first CF exacerbation, and a significant decrease in the bacterial genus *Parabacteroides* has been shown to precede the onset of chronic lung colonization by *Pseudomonas aeruginosa* [66]. Many additional human and animal studies have validated that alterations in the diversity and composition of bacteria within the gut of those with CF correlate with disease onset and severity [67].

One study suggests that these microbial alterations in the gut may also play an important role in the development of CFLD in susceptible individuals. The authors of this murine study found that diet may increase the risk of CF-associated cholangiopathy through alterations in the gut microbiota [68]. *Cftr*^−/−^ mice fed a high-triglyceride diet were found to have gut dysbiosis with increased relative abundance of *E. coli* and increased intestinal inflammation and permeability relative to *Cftr*^+/+^ mice fed the same diet. Ultimately, this group of mice developed features of cholangiopathy. They also had an increased liver-to-body weight ratio and mild portal fibro-inflammatory lesions on histology, which is consistent with liver damage. However, when *Cftr*^−/−^ mice were fed a diet containing polyethylene glycol (PEG), the abundance of intestinal *E. coli* was significantly lower compared to that of mice fed the high-triglyceride diet, and they had less intestinal inflammation and no evidence of liver damage.

Altogether, the findings of this preclinical study present compelling evidence for a role of gut dysbiosis in the pathogenesis of CFLD. This is also consistent with a human study that found alterations in the gut microbiota of patients with CFLD compared to CF patients without CFLD, such as decreased relative abundance of *Bacteroides* and increased abundance of *Clostridium*, correlating with increased gut inflammation [69]. It is important to examine these differences more closely in future studies, as both cohort studies and systematic reviews have linked the presence of CFLD with increased mortality in patients with CF [70,71].

### 2.5. Inflammatory Bowel Disease (IBD)

Pediatric inflammatory bowel disease (IBD) is a multifactorial condition that is characterized by chronic gastrointestinal inflammation and is associated with various genetic, environmental, and immune factors. Recent epidemiological studies describe an increasing incidence of pediatric IBD and very early-onset IBD (the presence of IBD symptoms arising prior to six years of age, associated with unique etiologies and clinical features); this underscores the importance of having a better understanding of how this condition affects other aspects of pediatric health, including liver and biliary function [72,73].

There is a strong association between IBD and primary sclerosing cholangitis (PSC), another chronic inflammatory disorder that affects the hepatobiliary system and is characterized classically by ‘onion-skin’ fibrosis of the bile duct. Two studies of pediatric patients with PSC found that over 70% of children in both cohorts had concomitant IBD, which was most often ulcerative colitis (UC) [74,75]. While a definitive link between these two conditions remains unclear, growing evidence suggests that increased exposure of cholangiocytes to gut-derived MAMPs as a result of IBD-related microbial dysbiosis and compromised gut barrier function may contribute to the immune-mediated bile duct injury seen in PSC [76]. A recent cross-sectional study examined the gut microbiota of pediatric patients (age 3–19) with PSC, UC, and concomitant PSC+UC [77]. Higher levels of Proteobacteria and a decreased *Firmicutes: Bacteroidetes* ratio—a known marker of dysbiosis often associated with IBD—were found in the PSC+UC group. In children under the age of 10, those with PSC+UC had significantly higher levels of *Escherichia-Shigella* compared to children of other comparison groups. In children over 10 with PSC+UC, there was a higher abundance of *Prevotella 9* and *Veillonella* and a lower abundance of *Lactobacillus.* The authors also demonstrated a positive correlation between the abundance of *Veillonella* and increased bilirubin and between the abundance of *Megasphaera* and higher GGT values, both markers of disease severity. Although this paper sampled only a small number of patients with concomitant PSC+UC (n = 7), these findings suggest an important relationship between microbial disruptions in IBD and biliary disease. Furthermore, one case study that followed a seven year old PSC-UC patient treated with oral vancomycin found that after 90 days of antibiotics, the patient had decreased mucosal inflammation, calprotectin, and GGT that correlated with antibiotic-mediated microbial changes [78]. Oral vancomycin treatment resulted in increased gut and oral microbial diversity along with decreased abundance of *Fusobacterium* and *Haemophilus* in the gut. Paradoxically, this patient had an increased abundance of *Veillonella* after treatment, which the authors attribute to *Veillonella’s* resistance to vancomycin. Although antibiotics are often thought of as contributors to dysbiosis, this study suggests that antibiotics may play a beneficial role in ‘resetting’ a gut microbiota disrupted by inflammatory conditions such as IBD and PSC.

Other common hepatobiliary manifestations of IBD in children include autoimmune hepatitis, cholelithiasis, and portal/hepatic vein thrombosis [79]. Additionally, IBD therapy itself may predispose patients to liver injury; sulfasalazine, glucocorticoids, methotrexate, and anti-TNF therapies may result in drug-induced liver toxicity while immunosuppressants commonly prescribed for IBD may make children more susceptible to viral hepatitis [80]. One retrospective study found signs of liver pathology in 21 out of 119 children with IBD, which were most commonly classified as PSC (19%), NAFLD (19%), and drug-induced liver disease (9%) [80]. However, there was no specific diagnosis made in 33% of these patients with serum-, imaging-, or histology-defined liver injury. In another study of pediatric IBD, episodes of elevated liver enzymes were classified as idiopathic in 87% of cases [81]. This uncertainty underscores the need for further investigation into mechanisms of IBD-associated liver injury. As seen in PSC and other hepatobiliary diseases discussed in this review, the gut microbiota may be a promising link. Specifically, the roles of *Veillonella* and *Haemophilus*, which have been found to positively correlate with disease severity in IBD, may be worthy of future study [82].

### 2.6. Hepatitis B Infection

Finally, viral infection is an important and potentially devastating etiology of hepatic disease in the pediatric population, and studies have demonstrated that the systemic response to viral infection is influenced by the gastrointestinal microbial landscape. Gut bacteria have been shown to modulate antiviral type I and type III interferon responses through signaling pathways involving both bacteria-derived ligands as well as bacterial metabolites [83]. Additionally, bacteria within the gastrointestinal tract appear to affect the efficacy of antiviral drugs as well as vaccines [84].

Hepatitis B virus (HBV) is a double-stranded DNA virus most often contracted by children through vertical transmission. An important immunological distinction between pediatric and adult HBV infection is that children, particularly neonates, are more likely to become chronic carriers of HBV; up to 90% of children infected within the first year of life go on to develop chronic infection [85,86]. One murine study found that while all adult mice cleared HBV by 5 weeks post infection (wpi), more than 50% of younger mice remained positive for hepatitis B surface antigen (HBsAg) at 17 wpi [87]. These younger mice had impaired anti-HBsAg antibody production and fewer hepatitis B core antigen (HBcAg)-specific IFN-γ-secreting splenocytes. Subsequent antibiotic treatment of adult mice suggested that these age-dependent differences in anti-HBV immune responses (including the antibody response) were in part driven by differences in the gut microbiota between the two age groups, with microbial signals from adult mice driving more robust immune responses to HBV in hepatic cells.

The importance of the gut microbiota in the immune response to HBV was corroborated by several studies in adults, which have found an increase in pro-inflammatory, LPS-secreting Gram-negative genera in patients with severe disease compared to those with milder disease [88]. It has also been shown that beneficial SCFA-producing bacteria, *Lachnospiraceae*, may reduce LPS secretion and translocation of bacteria in those with HBV, thus affecting the likelihood of progression to chronic HBV and hepatocellular carcinoma [89].

## 3. Discussion: Therapeutic Outlooks

Children are affected by a unique variety of liver disorders, ranging from diseases of hepatobiliary metabolism, such as Wilson’s disease and NAFLD, to structural pathologies, such as biliary atresia. While further research is required to investigate the reciprocal interactions between multifactorial pediatric liver diseases and the gut microbiota, it is clear that key interactions do exist. In some conditions, such as NAFLD and biliary atresia, animal models have shown that maternal factors influence the gut microbiota of offspring and contribute to the development of liver disease. In other cases, such as Wilson’s disease and CF, the metabolic effects of the primary liver disease result in gut dysbiosis that further potentiates systemic effects of the underlying disease through immune mechanisms. A better understanding of this gut–liver axis crosstalk may reveal new therapeutic targets to help slow the progression of chronic hepatobiliary diseases and improve overall outcomes in children.

Probiotics are one option for ‘resetting’ the dysregulated microbiota by introducing bacterial strains that are deficient in disease states or that have been associated with downstream anti-inflammatory effects. Potential therapeutic possibilities may include *Bacteroides* for infants at high risk of cholestasis and *Bifidobacterium* for children with early NAFLD, as some promising trials have already explored. Pediatric patients with several of the liver conditions discussed here may benefit more generally from probiotics designed to increase alpha diversity or influence microbial composition to more closely reflect that of the mature gut. Probiotics have in fact been trialed even in the premature neonatal population and have not generally been associated with increased negative outcomes such as death, sepsis, or meningitis [90,91]. However, this is a vulnerable population with an immature immune system, and there is evidence that the efficacy of probiotics varies with the disease targeted as well as the composition and quantity of bacteria within the supplement. Therefore, other therapeutic options may be safer and more efficacious.

Maternal diet modification during pregnancy is an attractive option to prevent the development of liver disease in the children of women with known risk factors. Such patients include women with type 2 diabetes mellitus (a maternal risk factor for BA) and obesity (a maternal risk factor for pediatric NAFLD) [92,93]. There are a number of studies that suggest that maternal diet can affect the neonatal gut microbiota as well as the overall neonatal immune environment by modulating the maternal microbiota and changing the nutrients available to the developing fetus [94,95]. For instance, a maternal high fat diet has been associated with decreased microbial diversity and relative reduction in *Bacteroides* in the neonatal GI tract [96,97]. Maternal zinc supplementation during pregnancy has been found to improve hepatitis B antibody response in neonates [98]. It is hypothesized that maternal diet may affect neonates by altering microbial colonization of the infant at birth or the placental transfer of microbial cytokines and immunoglobulins and dietary metabolites that differentially prime the fetal immune environment [94]. Because these mechanisms are independent of postnatal breastfeeding, this method of intervention has the added benefit of producing desired changes in neonates who are too premature or ill to receive enteral nutrition as well as neonates who are not breastfed.

Other potential interventions in pediatric patients include supplementation with prebiotics that promote the growth of beneficial bacteria or supplementation with postbiotics, the metabolites of bacteria. Such options for postbiotics may include butyrate or other SCFAs known to have global anti-inflammatory effects within the immune environment. However, further investigations are necessary to identify specific bacterial metabolites that influence the host response to inflammation, infection, and metabolic disruption in the setting of pediatric liver disease.

**Table 1:** Summary of clinical and preclinical findings on the gut–liver interactions in pediatric hepatobiliary diseases.

Condition	Clinical Findings	Preclinical Findings	Citations
Biliary Atresia (BA)/Cholestasis	Lower gut microbial diversity; decreased abundance of *Bifidobacterium*, *Bacteroides*, and *Faecalibacterium* (case-control study) Increased abundance of *Streptococcus* and *Klebsiella;* decreased abundance of *Bacteroides* and *Faecalibacterium* (case-control study) An infant with a good outcome following Kasai procedure for BA had higher alpha diversity; increased abundance of *Enterobacter*, *Bacteroides*, and *Clostridium* (case study)	Maternal supplementation with butyrate protected offspring from virally-induced BA; butyrate supplementation enriched *Firmicutes* and *Bacteroidetes* (murine study)	[26,28,29,30]
Pediatric Non-Alcoholic Fatty Liver Disease (NAFLD)	Children who became overweight by age 7 had higher levels of *Staph. aureus* and lower levels of *Bifidobacteria* in their stool during infancy compared to children who maintained a healthy weight (case-control study) A decrease in stool *Oscillospira* along with an increase in 2-butanone found to be a signature of pediatric patients with NAFLD; an increase in *Ruminococcus* and *Dorea* predicted NAFLD progression (case-control study) Children with NAFLD who received a probiotic for 3 months had decreased mean levels of alanine aminotransferase, aspartate aminotransferase, cholesterol, low-density lipoprotein C, triglycerides, and decreased waist circumference (RCT) Children with NAFLD who received a probiotic for 4 months had improved disease based on ultrasound findings compared to those who received placebo (RCT) Significant improvement in liver transaminases, total cholesterol, triglyceride levels, and ultrasound findings in pediatric NAFLD patients who received probiotics (meta-analysis)	Gnotobiotic mice gavaged with stool from infants born to mothers with obesity had increased weight gain, endoplasmic reticulum stress, liver macrophage accumulation, histologic evidence of increased periportal inflammation in the liver, and macrophage hypo-responsiveness to LPS	[40,44,46,47,48,49]
Wilson’s Disease	Compared to controls, patients with Wilson’s disease had lower gut microbial diversity; increased abundance of *Bacteroidetes;* lower abundance of *Firmicutes*, *Proteobacteria*, and *Fusobacteria;* decreased T lymphocyte populations; increased B lymphocytes, NK cells, and circulating levels of IgM (case-control study) Compared to controls, patients with Wilson’s disease had significantly lower abundance of *Firmicutes;* higher abundance of *Proteobacteria.* Microbial alteration may promote a pro-inflammatory environment within the gut (case-control study)	High-copper diet resulted in liver injury and gut barrier dysfunction in male rats stressed with a high-fructose diet; rats fed high-copper diet had an increased *Firmicutes: Bacteroidetes* ratio, enrichment of *Erysipelotrichaceae* and *Enterobacteriaceae*, and a reduction in *Akkermansia* Piglets with copper supplementation had increased *E. coli* in the gut; increased *E. coli* resistance to several antibiotics; altered microbial metabolic functions related to amino acid synthesis, energy metabolism, and protein metabolism	[53,54,55,56]
Cystic Fibrosis (CF)	Bacterial communities within the gut and lungs of infants with CF closely mirrored one another and shifted concordantly over time, with changes in diet resulting in changes in the lung microbiota (cohort study) Increased intestinal microbial alpha diversity found to positively correlate with increased time to infants’ first CF exacerbation; significant decrease in *Parabacteroides* preceded the onset of chronic lung colonization by *Pseudomonas aeruginosa* (cohort study) Compared to CF patients without liver disease, those with liver disease had decreased relative abundance of *Bacteroides* and increased abundance of *Clostridium*, correlating with increased gut inflammation (case-control study)	*Cftr*^−/−^ mice fed a high-triglyceride diet had gut dysbiosis, with increased relative abundance of *E. coli* and increased intestinal inflammation and permeability relative to *Cftr*^+/+^ mice fed the same diet; *Cftr*^−/−^ mice developed features of cholangiopathy. *Cftr*^−/−^ mice fed a polyethylene glycol diet had decreased abundance of intestinal *E. coli;* less intestinal inflammation; and no evidence of liver damage	[65,66,68,69]
Inflammatory Bowel Disease (IBD)	Higher levels of Proteobacteria and a decreased *Firmicutes: Bacteroidetes* in children with concomitant PSC + ulcerative colitis (UC) compared to children with just PSC or UC; children <10 with PSC+UC had higher levels of *Escherichia-Shigella*; children >10 with PSC+UC had a higher abundance of *Prevotella 9* and *Veillonella* and a lower abundance of *Lactobacillus*; positive correlation between the abundance of *Veillonella* and increased bilirubin and between the abundance of *Megasphaera* and higher GGT values (cross-sectional study) A 7 year old patient with PSC+UC treated with oral vancomycin for 90 days had decreased mucosal inflammation, calprotectin, and GGT that correlated with increased gut and oral microbial diversity; decreased abundance of *Fusobacterium* and *Haemophilus* in the gut (case study)		[77,78]
Hepatitis B Virus	In adult patients with HBV, there is an increase in pro-inflammatory, LPS-secreting Gram-negative genera in patients with severe disease compared to those with milder disease (review)	All adult mice infected with HBV cleared the virus by 5 weeks post infection (wpi), while >50% of younger mice remained positive for hepatitis B surface antigen (HBsAg) at 17 wpi; antibiotic administration decreased normal gut flora and prevented adult mice from clearing HBV as quickly	[87,88]

## Data Availability

No new data were created or analyzed in this study. Data sharing is not applicable to this article.
